# Effects of genetically modified maize events expressing Cry34Ab1, Cry35Ab1, Cry1F, and CP4 EPSPS proteins on arthropod complex food webs

**DOI:** 10.1002/ece3.2848

**Published:** 2017-03-08

**Authors:** Zoltán Pálinkás, József Kiss, Mihály Zalai, Ágnes Szénási, Zita Dorner, Samuel North, Guy Woodward, Adalbert Balog

**Affiliations:** ^1^ Institute of Plant ProtectionFaculty of Agriculture and Environmental SciencesSzent István UniversityGödöllőHungary; ^2^ Faculty of Natural SciencesDepartment of Life SciencesImperial College LondonLondonUnited Kingdom; ^3^ Department of HorticultureFaculty of Technical and Human ScienceSapientia Hungarian University of TransylvaniaCluj NapocaRomania

**Keywords:** characteristic path length, herbivores, non‐target organisms, predators, trophic groups

## Abstract

Four genetically modified (GM) maize (*Zea mays* L.) hybrids (coleopteran resistant, coleopteran and lepidopteran resistant, lepidopteran resistant and herbicide tolerant, coleopteran and herbicide tolerant) and its non‐GM control maize stands were tested to compare the functional diversity of arthropods and to determine whether genetic modifications alter the structure of arthropods food webs. A total number of 399,239 arthropod individuals were used for analyses. The trophic groups’ number and the links between them indicated that neither the higher magnitude of *Bt* toxins (included resistance against insect, and against both insects and glyphosate) nor the extra glyphosate treatment changed the structure of food webs. However, differences in the average trophic links/trophic groups were detected between GM and non‐GM food webs for herbivore groups and plants. Also, differences in characteristic path lengths between GM and non‐GM food webs for herbivores were observed. Food webs parameterized based on 2‐year in‐field assessments, and their properties can be considered a useful and simple tool to evaluate the effects of *Bt* toxins on non‐target organisms.

## 1 Introduction

The possible influence of genetically modified (GM) crops on biodiversity has been a topic of general interest for researchers and policy makers for some time. While several European countries have excluded the cultivation of GM crops or placed a moratorium on their release, others (especially USA, South American and Far East countries) are increasing both financial investment and land area dedicated to GM crops (Carpenter, 2011). The dominant GM crop globally is the GM maize; its cultivated area is now 35 million hectares in 11 countries worldwide (Bruinsma, 2015; Panel on Plant Protection Products and their Residues 2015). In the USA, from the total amount of maize cultivated in 2012, about 85% was genetically modified (Clive, 2012). According to the ISAAA (International Service for the Acquisition of Agri‐Biotech Applications) (Clive, 2012), the two main applications for GM maize at present are herbicide (mostly glyphosate) tolerance and pest (Coleoptera and/or Lepidoptera) resistance, or a combination of the two functions. However, non‐target arthropods may be exposed to *Bacillus thuringiensis* (*Bt*) endotoxins either by direct consumption of *Bt* plant material (including pollen) (Coll & Guershon, 2002), or by secondary consumption—feeding on prey that has previously ingested *Bt* protein (Harwood, Wallin, & Obrycki, 2005; Obrist, Dutton, Albajes, & Bigler, 2006). Several field and laboratory analyses have previously been made in both commercial and experimental fields to evaluate the potential impacts of GM crops containing transgenes from *Bt* on non‐target organisms (Romeis, Meissle, & Bigler, 2006). Most of these studies have focused on MON810 (Lepidoptera‐resistant GM maize), first commercially grown in 1996 (Balog, Kiss, Szekeres, Szénási, & Markó, 2010; Balog, Szénási, Szekeres, & Pálinkás, 2011; Naranjo, 2009; Romeis et al., 2006). All these assessments revealed no or transient effects on non‐target organisms when compared with non‐*Bt* controls (Romeis et al., 2006; Wolfenbarger, Naranjo, & Lundgren, 2008). Field studies have assessed the effects of GM exposure for many species (Raybould, 2006; Raybould, Stacey, & Vlachos, 2007), but arthropod food web analyses and food web topology comparisons between different GM and non‐GM maize hybrids have rarely been performed (Barratt, Todd, Burgess, & Malone, 2011; Mulder & Lotz, 2009; Szénási, Pálinkás, Zalai, Schmitz, & Balog, 2014). Consequently, there remains much unexplained variation in the structure and functioning of food webs and their parameters among systems. This is especially a central issue in analyses of insect communities associated with different host plant species (Squire, Hawes, Begg, & Young, 2009). Datasets with thousands of individuals can provide a useful tool in testing the possible effects of different GM maize strands on non‐target organisms (Szénási et al., 2014) and developing biosafety risk assumptions for arthropods exposed to GM plants (Barratt et al., 2011; Mulder & Lotz, 2009; Powell, 2007; Powell et al., 2009). Community studies must represent a detailed and multi‐methodological field assessment of all organisms from a GM and non‐GM crop over several years and throughout the growing season. Such datasets allow the functional diversity of the communities in differently treated ecosystems such as GM and non‐GM to be analyzed at the same time, providing consistent species abundance and prey preference data as food web parameters (Barratt et al., 2011; Mulder & Lotz, 2009; Powell et al., 2009; Szénási et al., 2014). During the present field assessment, detailed arthropod collections on both GM and non‐GM control maize were conducted (Table 1). Above‐ground food webs were then constructed using CoSBiLab software (Jordán, Gjata, Mei, & Yule, 2012), based on the abundance of, and interactions between, different trophic groups. Trophic groups were defined as groups of taxa that share the same food preference within a food web (Dunne, Williams, & Martinez, 2002). For example, in this investigation ground beetles (Carabidae) and spiders both feed on Alticinae and Collembola).

**Table 1:** Properties of the GM and non‐GM maize stands investigated in Sóskút, Hungary, 2007–2008. Treatment coding (1, 2, 5, 6, 7, 8, 901, and 903) approved by the Pioneer Genetique were followed

Treatm.	OECD Ident.	Resistance	Toxins	no. of repl.
1	DAS‐59122‐7	Coleoptera	Cry34Ab1, Cry35Ab1	4
2	DAS‐01507‐1xDAS‐59122‐7	Coleoptera and Lepidoptera	Cry34Ab1, Cry35Ab1xCry1F	4
5	DAS‐01507‐1xMON‐00603‐6	Lepidoptera and glyphosate	Cry1FxHT	4
6	DAS‐01507‐1xMON‐00603‐6	Lepidoptera and glyphosate + glyphosate treatment	Cry1FxHT	4
7	DAS‐59122‐7xDAS‐01507‐1xMON‐00603‐6	Coleoptera, Lepidoptera and glyphosate	Cry34Ab1, Cry35Ab1xCry1FxHT	4
8	DAS‐59122‐7xDAS‐01507‐1xMON‐00603‐6	Coleoptera, Lepidoptera and glyphosate + glyphosate treatment	Cry34Ab1, Cry35Ab1xCry1FxHT	4
901	PR‐36B08	NO	Control	4
903	PR‐35Y65	NO	Control	4

We hypothesized that the above‐ground food webs and associated metrics (e.g., average trophic links/trophic groups ‐ a complexity factor that considers the number of trophic groups and number of links between them, and “characteristic path length”—a measure of the efficiency of consumption in a food web (the mean shortest food chain length) counted shortest path length between two trophic groups averaged over all pairs of groups) in GM and non‐GM maize can be a useful tool to compare the functional diversity (i.e., due to indirect effects by reduce prey resulting in reduced predators) of the arthropods and to evaluate the effects of different GM crops on arthropod communities. Here, a total of almost 400,000 individuals, with an average of 25,000–34,000 arthropod individuals/treatment, were used to construct food webs. To test the potential cascading effects of toxins (*Bt* and/or glyphosate in this case) in arthropod communities, food webs and its parameters represent high interest from consumers, scientists, and stakeholders.

## 2 Material and Methods

### 2.1 Ethics statement

No permits were required for field assessment and collections of arthropod. All animal work was led according to related national and international guidelines (European Commission, Ethics for Researchers 2013).

### 2.2 Study sites

Arthropods collections were conducted over 2 years (2007 and 2008) in the maize growing seasons (early‐June, late‐September) in an experimental field specifically designed for this experiment and surrounded by peach and apricot orchards (for GM stands isolation as required by authorities) in west of Budapest, Hungary (47 25°N, 18 47°E) (Figure S1, Supporting Information).

### 2.3 GM and non‐GM maize stands used for assessment

Altogether four GM maize hybrids and two isogenic (non‐GM) controls were used as follows.

1. Coleoptera‐resistant GM maize stand,
2. Coleoptera and Lepidoptera resistant GM maize stand,
3. Lepidoptera and glyphosate tolerance GM maize stand,
4. Coleoptera and Lepidoptera resistant and glyphosate tolerance GM maize stand,
5. Non‐GM maize control PR‐36B08,
6. Non‐GM maize control PR‐35Y65.

All GM stands with insect resistance and non‐GM controls were replicated four times (four blocks), each block area was 625 m^2^ (25 × 25 m). The two GM maize hybrid stands with glyphosate tolerance were however replicated randomly eight times (eight blocks), from which four randomly selected blocks were additionally treated with an extra glyphosate herbicide (spraying mechanically with 1060 g/ha of glyphosate in each year ‐ entries 6 and 8) (Table 1, Figure S1, Supporting Information).

### 2.4 Proteins expression by GM maize hybrids

Maize stands with Coleoptera resistance contains two components: 1. *Cry34Ab1*, a 14‐kDa protein, and 2. *Cry35Ab1*, a 44‐kDa protein, the combination of which forms a binary toxin active ingredient against western corn rootworm (*Diabrotica virgifera virgifera* LeConte) larvae feeding on maize roots. Maize stands with Lepidoptera contains TC1507 express *Cry1F* protein against the European corn borer larvae (*Ostrinia nubilalis* Hubner) feeding inside the maize stalk between internodes. Maize stands with glyphosate tolerance express the CP4 EPSPS protein, which confers resistance (tolerance) to glyphosate herbicides (Table 1).

### 2.5 Cropping procedures

An alley distance of 3 m (for isolation and for mechanical intervention—harvest at the end of the experiment) was used between blocks. A non‐GM maize hybrid of similar maturity as the tested groups was planted around the entire study field for pollen capture. This was done in order to prevent GM maize pollen from spreading to further distance (Supporting Information, Figure S1). This prevention maize zone has not been included in the experiment (no collections were made here). All GM and non‐GM hybrids were seeded between late‐April and mid‐May and were harvested between mid‐October and early‐November in both years. No seed treatment was applied prior to plantation. The experimental plots (except those with extra glyphosate treatments) were not subject to any other type of pest management.

### 2.6 Arthropod sampling procedures

For 2 years (2007–2008) arthropods and weeds were collected and assessed weekly starting from June and until the end of September. Migration of arthropods between sites was very likely, thereby influencing the effects of the different treatments. To reduce this bias arising from plot size, arthropods were only collected from a 10 × 10 m extent area in the middle of each 625 m^2^ block. In this way a greater distance (and low possibility of influence) between traps of different replicates was achieved. Three standard methods (previously described by Szénási et al., 2014) were followed to collect arthropods:

1. Pitfall traps with ethylene‐glycol 10% were used to collect soil surface active (above‐ground) arthropods. Three traps in each block (a total of 96) were placed in the middle of each plot in triangle 10 m from each other and emptied weekly. All individual arthropods captured were transferred to the laboratory, identified, counted, and categorized into a specific trophic group.
2. Plant canopy dwelling arthropods were collected weekly by using 30 × 20 cm Pherocon^AM^ yellow sticky traps. Three traps/block (a total of 96) were used. Traps were changed every week from May until October. New traps were placed on the same plants, while the collected ones were transferred to the laboratory where all arthropods were counted, identified, and classified into trophic groups.
3. Assessments of the arthropods (i.e., several aphid species) where no trapping could be used due to lack of color sensitivity, were made by visual observations in each year of the sampling period. Fifteen plants per blocks were randomly selected in each week (480 plants per assessment) and all arthropods observed (morning between 10.00 to 14.00 hr) were counted, identified, and classified into trophic groups. Counted plants were marked and others were chosen for the next assessment, to avoid the possibility of recounting the same colonies.

Weed species from each block and their coverage (% covered by weeds from soil surface in a randomly selected 1 × 1 m^2^ areas) were assessed by visual survey in three random 1 × 1 m^2^ areas within the center of each plot.

### 2.7 Data analysis

First, all collected arthropods of similar food preferences, with at least 100 individuals/treatment/sample events/plots for herbivores and 20 individuals for predators, were categorized into trophic groups. Trophic groups were defined as functional groups of taxa with similar dietary preference within a food web. Authorities to define dietary preferences are given in Supporting Information, Table S1. This method is accepted in food web analyses and reduces methodological biases associated to uneven resolution of taxa within food webs (Jordán et al., 2012; Layer, Hildrew, & Woodward, 2013). Three key factors were first considered and data adjusted according to these in order to increase the sensibility of food webs: (1) Because maize pollen can travel between GM and non‐GM block as a result of wind activity, this may resulted in producing some kernels with *Bt* toxins in non‐*Bt* plots. Therefore food web construction associated with each GM and non‐GM maize was done using only data collected before pollen spreading (mid‐June to mid‐July). (2) Seasonal changes in abundance of arthropods were an important consideration too. For example, western corn rootworms adults are not present in April, May, June, September, October, and November, but are present in July and August (Panel on Genetically Modified Organisms 2010). Therefore, data for food web construction were only considered when the abundance of the dominant trophic groups were the highest, but prior to pollen spreading. (3) Food webs were constructed for each individual plot, and similar replicates of each treatment compared. As no differences in food webs within treatments of GM and non‐GM maize were detected (similar number of trophic groups and link between them were recorded), food webs of the same treatments were reconstructed by pooling and averaging the data of the similar replicates.

**Figure 1:**
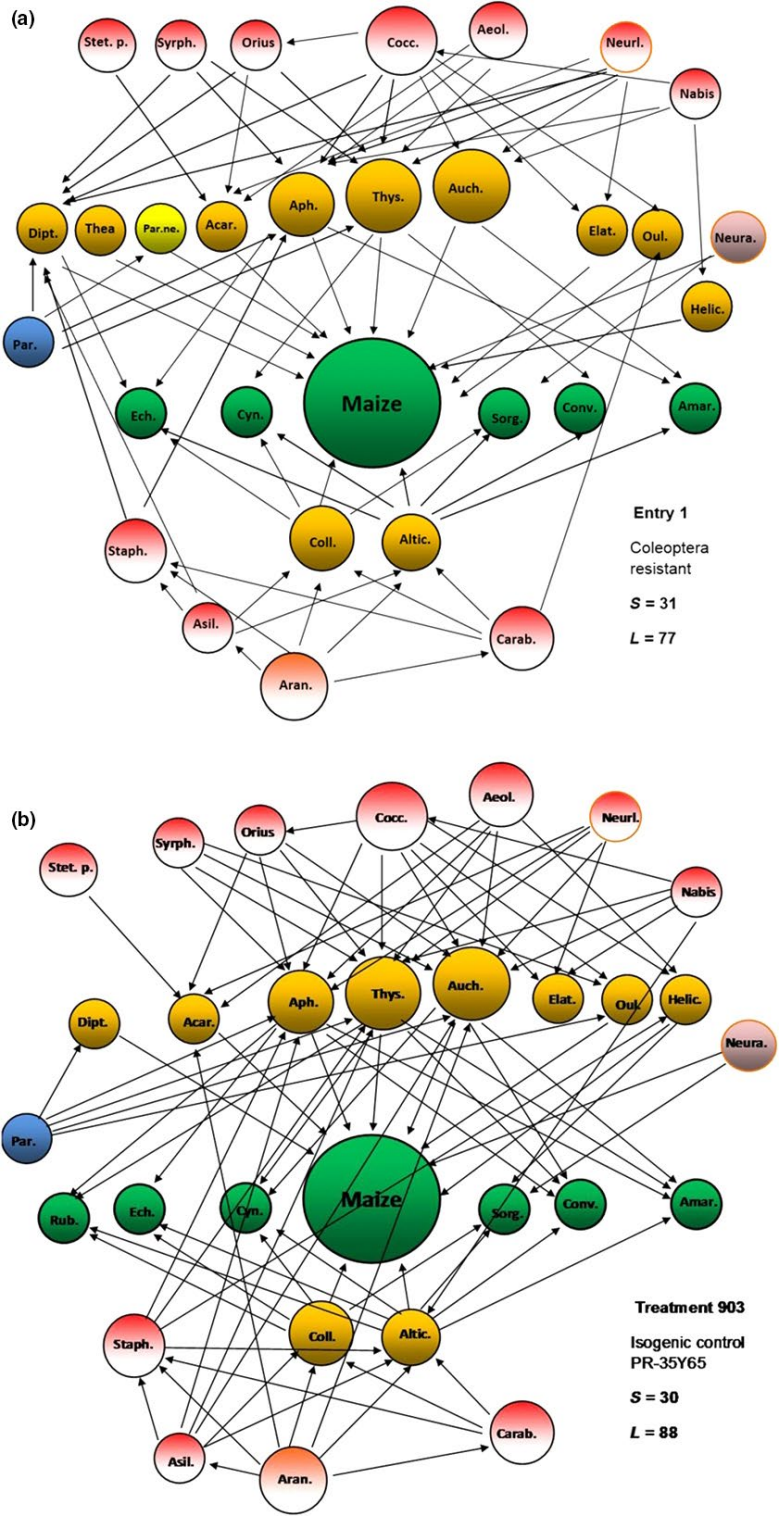
(a, b) Food web in Coleoptera‐resistant maize (Treatment 1) (a) and non‐GM control maize (Treatment 903) (b), built by considering the abundance and interactions between trophic groups. The position and size of the circles intended to allow an easier way to identify functional groups. Figures are graphically adjusted by software (circles sizes) with abundances and were intended to allow an easier way to identify functional groups. Green circles, vegetation; orange circles, herbivores; red circles, predators; blue circle, parasitoids; yellow circle, parasitized neuropteran egg. *S*, number of trophic groups; *L*, number of links between trophic groups. Food webs were built with CoSBiLab software (Jordán et al., 2012). Weed species: Ech., *Echinocloa crus‐galli*; Cyn., *Cynodon dactylon*; Sorg., *Sorghum halapense*; Conv., *Convolvulus arvensis*; Amar., *Amaranthus retroflexus* (the sixth weed species for entries 5, 8 and 903 was Rub., *Rubus caesius*). Herbivore group: Acar., *Acaridae*; Coll., *Collembola*; Aph., *Aphididae*; Auch., *Auchenorrhyncha*; Thys., *Thysanoptera*; Altic., *Alticinae*; Elat., *Elateridae*; Oul., *Oulema melanopus*; Thea, *Thea vigintiduopunctata* (powdery mildew consumer); Dipt., *Diptera*; Helic., *Helicoverpa armigera*; Predatory group: Stet. p., *Stethorus punctillum* (mite predator ladybeetle adult and larvae); Cocc., *Coccinellidae* (all polyphagous species); Neura., *Neuroptera* adult; Neurl., *Neuroptera* larvae; Aeol., *Aeolothripidae*; Carab., *Carabidae*; Staph., *Staphylinidae*; Nabis, *Nabis* spp.; Ori. , *Orius* spp.; Asil., *Asilidae* (adults and larvae); Syrph., *Syrphidae*; Aran., *Araneae*; Par., Parasitoids; Par. ne., Parasitized neuropteran egg

Before food webs were constructed, predator–prey interactions were searched in scientific literature and prey preferences checked (Supporting Information, Table S1). We collated trophic interactions among each species within trophic groups (or the next highest level of resolution available, usually genus), from 62 different scientific literary sources (Supporting Information, Table S1). The taxonomy of every resource and consumer has been standardized through the Global Names Resolver (http://resolver.globalnames.biodinfo.org/) using the Global Biodiversity Information Facility dataset (Gray et al., 2014). The food web of each GM treatment and its controls were constructed using CoSBiLab software (Gagic, Tscharntke, & Dormann, 2011; Jordán et al., 2012) based on trophic groups abundance and trophic interactions among nodes. The food web constructions include below level maize and all dominant weed species followed by herbivore groups, predators and parasitoids present in both 2 years of assessment (Figure 1a,b). To test how the generated food webs compared to their empirical counterparts, the following network metrics were calculated (Christian & Luczkovich, 1999; Goldwasser & Roughgarden, 1993; Martinez, Hawkins, Dawah, & Feifarek, 1999 *)*: Average trophic link/trophic groups (*B* = *L*/*S*)—This considers the number of trophic groups (*S*) and number of links (*L*) between them (Albert & Barabási, 2002; Barabási & Albert, 1999). Characteristic path length (*D* = 2/*N*(*N* − 1)∑*d*_*ij*_)—the shortest path length between two trophic groups averaged over all pairs of groups (Albert & Barabási, 2002; Antoniou & Tsompa, 2008; Barabási & Albert, 1999; Dunne et al., 2002; Jordán et al., 2012). The average trophic link/trophic group (*B*) and characteristic path length values (*D*) for each trophic level were considered in statistics as the mean of four replicate plots of each treatment. A paired *t* test was used to compare *B* and *D* values for each trophic level in the GM treatments to the average of the values in the non‐GM (control) treatments.

## 3 Results

The food webs were constructed from approximately 25,000 to 34,000 arthropod individuals per treatment during the 2‐year period, with a total number of 399,239 individuals analyzed from 32 plots (24 GM and eight non‐GM). The most abundant herbivores in all treatments were aphids (Aphididea), thrips (Thysanoptera), leafhoppers (Cicadellidae), flea beetles (Alticinae) and mites (Acaridae). The most important predatory groups were spiders (Araneae), dumsel bugs (*Nabis* spp.), ladybeetles (Coccinellidae), ground beetles (Carabidae), rove beetles (Staphilinidae), lacewing larvae (Neuroptera) and minute pirate bugs (*Orius* spp.) (Figure 1a,b). The structure of interactions between trophic groups showed low or no changes across GM and non‐GM crops (Supporting Information, Figure S2A–G). The vegetation composition (not the density) and the structure of herbivores as well as predatory groups were similar in all entries and presented high interactions diversity and linkage density (Figure 1a,b). Food web analyses revealed a relatively constant number of trophic groups (*S*) that varied between 30 and 33 (Figure 1a,b). All food webs consisted of a relatively uniform directed trophic links (*L*) between trophic groups that varied between 77 and 95 (Supporting Information, Figure S2A–G). The average trophic link/trophic groups (*B*) varied between 4.5 and 5.5 for predators, between 5.14 and 6.14 for herbivores and between 5.43 and 5.67 for vegetation (Table 2). Characteristic path length (*D*) values for predators varied between 0.16 and 0.21, for herbivores between 1 and 1.21 and between 1.71 and 2 for vegetation (Table 2). All these data demonstrate that the efficiency of consumption in a food web is high. Statistical comparison between GM and non‐GM maize food web parameters (average trophic link/trophic groups (*B*) and characteristic path length (*D*)), using paired *t* test demonstrated differences in the average trophic link/trophic groups for both herbivore groups and vegetation. Differences in characteristic path lengths between GM and non‐GM food webs for herbivores were also observed (Table 2).

**Table 2:** Food web parameters of GM and non‐GM maize food webs based on the total arthropods collected during an extensive 2 year in‐field assessment

Tre.	Resistance				Control 901 PR‐36B08		Control 903 PR‐35Y65			Control 901 PR‐36B08		Control 903 PR‐35Y65	
		*S*	*L*	*B*	*t*	*p*	*t*	*p*	*D*	*t*	*p*	*t*	*p*
		Predators											
1	Col.	12	54	4.50	−2.07	.06	−1.67	.11	0.23	0.91	.38	0.71	.49
2	Col. and Lep.	12	55	4.58	−1.92	.07	−1.41	.16	0.22	0.90	.38	0.70	.49
5	Lep. and glyph.	13	59	4.54	−1.74	.1	−1.36	.19	0.20	0.53	.60	0.28	.77
6	Lep. and glyph.+ glyph. tr.	12	60	5.00	−1.96	.06	−1.60	.13	0.20	0.53	.60	0.28	.77
7	Col., Lep. and glyph.	12	66	5.50	−1.64	.11	−1.31	.20	0.16	−0.16	.87	−0.51	.61
8	Col., Lep. and glyph. + glyph. tr.	13	66	5.08	−1.99	.06	−1.65	.11	0.16	−0.16	.87	−0.51	.61
		Herbivores											
1	Col.	13	70	5.38	−2.81	**.00**	−2.72	**.00**	1	−2.74	**.01**	−2.32	**.03**
2	Col. and Lep.	14	72	5.14	−2.92	**.00**	−2.83	**.00**	1.02	−2.55	**.02**	−2.13	**.05**
5	Lep. and glyph.	14	79	5.64	−2.56	**.00**	−2.47	**.01**	1.04	−2.68	**.01**	−2.31	**.03**
6	Lep. and glyph.+ glyph. tr.	14	76	5.43	−3.21	**.00**	−3.11	**.00**	1.15	−2.57	**.02**	−2.18	**.04**
7	Col., Lep. and glyph.	14	82	5.86	−2.74	**.00**	−2.66	**.00**	1.21	−2.88	**.01**	−2.52	**.02**
8	Col., Lep. and glyph. + glyph. tr.	14	86	6.14	−2.68	**.01**	−2.60	**.01**	1.17	−3.54	**.00**	−3.17	**.00**
		Vegetation											
1	Col.	6	34	5.67	−3.04	**.00**	−3.54	**.00**	1.71	0.01	.98	−0.28	.35
2	Col. and Lep.	6	34	5.67	−3.04	**.00**	−3.54	**.00**	1.71	0.01	.98	−0.28	.35
5	Lep. and glyph.	7	38	5.43	−3.04	**.00**	−3.57	**.00**	2	0.01	.98	0.01	.98
6	Lep. and glyph.+ glyph. tr.	6	34	5.67	−3.58	**.00**	−4.14	**.00**	1.71	0.01	.98	−0.28	.35
7	Col., Lep. and glyph.	6	34	5.67	−3.04	**.00**	−3.54	**.00**	1.71	0.01	.98	−0.28	.35
8	Col., Lep. and glyph. + glyph. tr.	6	38	6.33	−3.04	**.00**	−3.57	**.00**	2	0.01	.98	0.01	.98

*S*, number of trophic groups; *L*, number of links between trophic groups; *B,* trophic links/group; *D*, characteristic path length.

Bolded “*p*” values represent statistical significant differences. Paired *t*‐test was used to compare *B* and *D* values for each trophic level in the GM treatments to the average of the values in the non‐GM treatments.

## 4 Discussion

Despite the total number of arthropods analyzed in this exceeding 399,000, the outcome of the results only represent a short‐term effect of GM maize on arthropod food webs. The study revealed that food webs with a high number of interacting groups co‐exist in both GM and non‐GM maize (Figure 1a,b, Supporting Information, Figure S2A–G). The study also revealed that possible influence of *Bt* toxins on non‐target arthropods can be analyzed simultaneously through the use of food webs and its properties. This method also makes possible simple comparisons between GM and non‐GM maize strands through vertical interactions of trophic groups. All key trophic groups detected in both herbivores (aphids, thrips, Colembola, Alticinae) and predators (ladybirds, spiders, ground beetles, rove beetles) were identified in previous studies as non‐target organisms which might be vulnerable to possible deleterious effects of *Bt* toxins, both directly (herbivores) and indirectly (predators) (Balog et al., 2010, 2011; Harwood et al., 2005; Lundgren & Duan, 2013; Meissle & Romeis, 2009; Obrist et al., 2006; Szénási et al., 2014). There is no concrete evidence that *Bt* toxins accumulate in prey tissues (Carstens, Anderson, & Bachman, 2012; Romeis et al., 2006). Individual analyses in other studies have also revealed no detrimental effects on arthropod predators (studies on lacewing larvae and lady beetles under laboratory conditions (Romeis et al., 2006), adult and juvenile *Theridion impressum* (Koch) spiders (Meissle & Romeis, 2009), open field and laboratory studies on *Orius* spp., lacewings and *Stethorus punctillum* (Weise) (Meissle & Romeis, 2009; Obrist et al., 2006), Staphylinidae (Farinós, de la Poza, & Hernández‐Crespo, 2008) and Lepidoptera (Schuppener, Mühlhause, Müller, & Rauschen, 2012). There were also no significant variations in Heteropteran predator abundances exposed with herbicide (glyphosate)‐tolerant maize varieties (Albajes, Lumbierres, & Pons, 2011). Prey‐specialist predatory arthropods (e.g., parasitoids) that rely more closely on target pests may be the exception to the generalization that *Bt* maize does not impact negatively non‐target organisms. This is predominantly true for specialist parasitoid species, which abundances are likely to decrease along with their herbivorous hosts (Hellmich et al., 2008). The present study (does not identify specific host and only for non‐target organisms), however, revealed that parasitoids were present in relatively high abundance even in all GM and non‐GM maize with relatively high connectivity (Figure 1a,b). According to the results, the extra application of the glyphosate reduced the overall weed density; broadleaf weeds were, however, still able to persist in low mass because of previously spread and sprouted seeds in soil. This post‐herbicide application weed density was an important food source for herbivores. The average trophic link/trophic groups (*B*) do not necessarily increase if node number increases in a food web. Differences, however, in the average trophic link/trophic groups were detected between GM and non‐GM food webs for herbivore groups and for vegetation (Table 2). This can be explained again with altered weed densities in all GM crops with about 10% (Coleoptera resistant) to 30% (glyphosate resistant). Higher path length values (>4) means that the food web is linear, while lower values indicate that the food web has a stable compact form (Albert & Barabási, 2002; Antoniou & Tsompa, 2008; Dunne et al., 2002; Jordán et al., 2012). Differences in characteristic path lengths show differences in consumption efficiency in food web between GM and non‐GM crops for herbivores (Table 2). This again can be explained with altered weed densities in GM crops that influences herbivore‐weed connections (a total of 28 connections to plants from which 13 to maize in treatment 1 with Coleoptera resistance, and 33 connections to plants from which 10 to maize in control 903; Figure 1a,b). The higher herbivore—maize connections in all GM treatments may suggest that GM maize hybrids do not detrimentally alter arthropod food webs. Altogether, we can conclude that the realistically parameterized food webs and their properties can be considered a useful tool to evaluate the potential detrimental effects of *Bt* toxins on non‐target organisms and to investigate and compare the functional diversity of arthropods on GM and non‐GM maize strands.

## Conflict of Interest

Authors declare no conflict of interest. All authors express their thanks to Pioneer Genetique for providing the maize seed materials for research.

## Authors Contributions

Z.P., M.Z., Á.Sz., and Z.D. performed the experiment, S.N., G.W., and A.B. wrote the main manuscript text. A.B., J.K., and Z.P. prepared figures and tables. All authors reviewed the last version of the manuscript and were agree with submission.
