# Supplementary material for: Effects of genetically modified maize events expressing Cry34Ab1, Cry35Ab1, Cry1F, and CP4 EPSPS proteins on arthropod complex food webs
Source: Ecol Evol. 2017 Mar 8;7(7):2286–93. doi: 10.1002/ece3.2848 (PMC5383485; doi:10.1002/ece3.2848)
Supplement: Supplementary file 1 [file ECE3-7-2286-s001.docx]

**Supplementary online material**

**
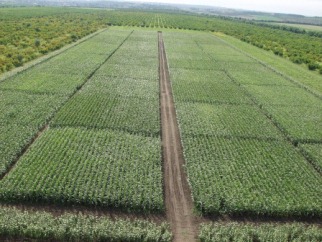
**

**Figure S1.** The aerial view of the investigated GM and non-GM maize stands (picture made by Prof. József Kiss).

**Table S1.** Trophic interactions among each species within trophic groups (or the next highest level of resolution available, usually genus) from scientific literary sources.

| **Predators** | **Prey species in maize fields** | **References** |
| --- | --- | --- |
| *Coleoptera*  *Adalia bipunctata*  (two-spot ladybird) | Generalist aphid predator | 1. Tirry JL, De Clercq P. (2009) Food consumption and immature growth of *Adalia bipunctata* (Coleoptera: Coccinellidae) on a natural prey and a factitious food. Eur. J. Entomol. 106: 193–198. 2. Vanhaelen N, Gaspar C, Francis F. (2002) Prey suitability as environmental effect on *Adalia bipunctata* reproduction. Meded Rijksuniv Gent Fak Landbouwkd Toegep Biol Wet. 67(3):563-8. 3. Blackman RL. (2008) Selection of aphid prey by *Adalia bipunctata* L. and *Coccinella 7- punctata* L. Ann. Appl. Biol. 59(3):331 – 338. |
| *Coleoptera*  *Adalia decempunctata*  (ten-spotted ladybird) | Generalist aphid predator hunts in trees and shrubs | 1. Dixon AFG. (1959) An Experimental Study of the Searching Behaviour of the Predatory Coccinellid Beetle *Adalia decempunctata* (L.) J. Anim. Ecol. 28(2):259. 2. Hodek I, Honek A, van Emden HF. (2012) Ecology and Behaviour of the Ladybird Beetles (Coccinellidae). Wiley-Blackwell. 600 pp. 3. Bellows TS, Fisher TW, Caltagirone LE, Dahlsten DL, Gordh G. Huffaker CB. (1999) Handbook of Biological Control - Principles and Applications of Biological Control. Elsevier. 1046 pp. |
| *Coleoptera Coccinella septempunctata* (seven-spot ladybird) | Generalist aphid predator, Tetranichus urticae (spider mites), Cicada, Lema melanopus | 1. Khan MH, Khan MR. (2002) Prey Preference and Switching Behaviour of *Coccinella septempunctata* L. (Coleoptera: Coccinellidae) International J Agric. Biol. 1560–8530/04–1–110–112. 2. Lucas E, Coderre D, Vincent C. (1997) Voracity and feeding preferences of two aphidophagous coccinellids on *Aphis citricola* and *Tetranychus urticae* Ent. Exp. Appl. 85: 151–159, 1997. 3. Obrycki JJ., Orr CJ. (1990) Suitability of Three Prey Species for Nearctic Populations of *Coccinella septempunctata*, *Hippodamia variegata*, and *Propylea quatuordecimpunctata* (Coleoptera: Coccinellidae). J. Econ. Entomol. 83(4):1292-1297. |
| *Coleoptera Coccinella quatuordecimpunctata*  (14-spotted Ladybird) | Generalist aphid predator | 1. Obrycki JJ., Orr CJ. (1990) Suitability of Three Prey Species for Nearctic Populations of *Coccinella septempunctata, Hippodamia variegata*, and *Propylea quatuordecimpunctata* (Coleoptera: Coccinellidae). J. Econ. Entomol. 83(4):1292-1297. 2. Kalushkovi, P. Hodek I. (2005) The effects of six species of aphids on some life history parameters of the ladybird *Propylea quatuordecimpunctata* (Coleoptera: Coccinellidae). Eur. J. Entomol. 102 (3): 449-452. 3. Dixon AFG. (2000) Insect Predator-Prey Dynamics: Ladybird Beetles and Biological Control. Cambridge University Press. 226 pp. |
| *Coleoptera Hippodamia tredecimpunctata*  (thirteen-spot ladybird) | Generalist aphid predator | 1. Schellhorn NA., Andow DA. (2005) Response of coccinellids to their aphid prey at different spatial scales. Popul. Ecol. 47(1): 71-76. 2. Swaminathan R. Meena A. Meena, BM. (2015) Diversity and predation potential of major aphidophagous predators in maize. Appl. Ecol. Env. Res. 13(4): 1069-1084. 3. Wright EJ., Laing JE. (1980) Numerical response of coccinellids to aphids in corn in southern Ontario. Canadian Entomol. 112(10): 977-988. |
| *Coleoptera Hippodamia variegate* (variegated lady beetle) | Generalist aphid predator | 1. Dehkordi SD, Sahragard A (2013) Functional Response of *Hippodamia variegata* (Coleoptera: Coccinellidae) to Different Densities of Aphis gossypii (Hemiptera: Aphididae) in an Open Patch Design. J. Agr. Sci. Tech. 15: 651-659. 2. Shahaz MA, Khanb AA. (2014) Qualitative and Quantitative Prey Requirements of two Aphidophagous Coccinellids, *Adalia tetraspilota* and *Hippodamia variegate*. J Insect Sci. 2014; 14: 72. 3. Kontodimas DC., Stathas GJ. (2005) Phenology, fecundity and life table parameters of the predator *Hippodamia variegata* reared on *Dysaphis crataegi.* BioControl 50(2): 223-233. |
| *Coleoptera Stethorus punctillum adult and larvae*  (spider mite lady beetle) | Specialist Spider Mite Predator | 1. Alvarez-Alfageme F, Ferry N, Castañera P, Ortego F, Gatehouse AM. (2008) Prey mediated effects of Bt maize on fitness and digestive physiology of the red spider mite predator *Stethorus punctillum* Weise (Coleoptera: Coccinellidae). Transgenic Res. 17(5):943-54. 2. Lundgren JG. (2009) Book Review - Relationships of Natural Enemies and Non-Prey Foods Progress in Biological Control. Springer. 453 pp. 3. Sweet J. Bartsch D. (2012) Synthesis and Overview Studies to Evaluate Existing Research and Knowledge on Biological Issues on GM Plants of Relevance to Swiss Environments. Hochschulverlag AG an der ETH Zürich. |
| *Coleoptera Carabidae*  (ground beetles) | Generalist predators: other ground dwelling species like Phylotreta sp. Colembola, Staphylinidae adult and larvae, Spodoptera littoralis caterpilars. European Corn Borer | 1. Meissle M. Vojtech E. Poppy GM. (2005) Effects of Bt maize-fed prey on the generalist predator *Poecilus cupreus* L. (Coleoptera: Carabidae) Transgenic Res. 14(2): 123-132. 2. Kocoure FK , Saska P. Řezáč M. (2013) Diversity of Carabid Beetles (Coleoptera: Carabidae) under Three Different Control Strategies against European Corn Borer in Maize. Plant Protect. Sci 49(3): 146–153. 3. Balog A., Markó V., Szarvas P. (2008) Dominance, Activity Density and Prey Preferences of Rove Beetles (Coleoptera: Staphylinidae) in Conventionally Treated Agro-Ecosystems. Bull. Ent. Res. 98: 259-269. 4. Balog A., Szénási Á., Szekeres D., Kiss J. (2010) Staphylinids (Coleoptera: Staphylinidae) in genetically modified maize ecosystems: species densities and trophic interactions. IOBC-Bulletin, 52: 9-15. 5. Balog A., Kiss J., Szekeres D., Szénási Á., Markó V. (2010) Rove beetle (Coleoptera: Staphylinidae) communities in transgenic Bt (MON810) and near isogenic maize. Crop Prot. 29: 567-571. |
| *Coleoptera Staphylinidae*  (rove beetles) | Generalist predators, the diet includes aphids, spider mites, on plants but also eggs or other species even predtors larvae on ground. | 1. Balog A., Szénási Á., Szekeres D., Kiss J. (2010) Staphylinids (Coleoptera: Staphylinidae) in genetically modified maize ecosystems: species densities and trophic interactions. IOBC-Bulletin, 52: 9-15. 2. Balog A., Kiss J., Szekeres D., Szénási Á., Markó V. (2010) Rove beetle (Coleoptera: Staphylinidae) communities in transgenic Bt (MON810) and near isogenic maize. Crop Prot. 29: 567-571. 3. Balog A., Szénási A., Szekeres D., Pálinkás Z. (2011) Analysis of soil dwelling rove beetles (Coleoptera: Staphylinidae) in cultivated maize fields containing the Bt toxins, Cry34/35Ab1 and Cry1F x Cry34/35Ab1. Biocontrol Sci. Tech. 21: 3, 293-297. |
| *Diptera Asilidae adult*  (robber fly) | Other flies, beetles, butterflies and moths, various bees, ants, dragon and damselflies, ichneumon wasps, grasshoppers, and spiders. | 1. Richards OW., Davies RG. (1977) Imms' General Textbook of Entomology: Volume 1: Structure, Physiology and Development Volume 2: Classification and Biology. Berlin: Springer. ISBN 0-412-61390-5. 2. Musso JJ. (1983) Nutritive and ecological requirements of robber flies (Diptera: Brachycera: Asilidae). Entomologia Generalis 9: 35–50. 3. Dennis J., Lavigne, D. (1975) Comparative behavior of Wyoming robber flies II (Diptera, Asilidae) University of Wyoming Agricultural Experiment Station Science Monograph 30:1-68. 4. Predator-Prey Database for the family Asilidae (Hexapoda: Diptera). http://www.geller-grimm.de/catalog/lavigne.htm. |
| *Diptera Asilidae larvae*  (robber fly) | Generalist egg and pupae predators of other insects in the soil | 1. Musso JJ. (1983) Nutritive and ecological requirements of robber flies (Diptera: Brachycera: Asilidae). Entomologia Generalis 9: 35–50. 2. Jervis MA. (2005) Insects as Natural Enemies. A Practical Perspective. Springer. 748 pp. 3. Mugo S., Songa J., DeGroote H., Hoisington D. (2002) Insect Resistant Maize for Africa (IRMA) Project: An overview. IRMA Project (overview). Syngenta Symposium, June 25 2002 Washington DC. |
| *Diptera Syrphidae larvae*  (hoverflies) | Generalist aphid predators, feed with aphids on higher plants also occurring on shrubs and tall herbs. Also prey on thrips. | 1. Primante C., Dotterl S. (2010) A syrphid fly uses olfactory cues to find a non-yellow flower. J. Chem. Ecol. 36: 1207–1210. 2. Bischoff M., Campbell DR. Lord JM., Robertson AW. (2013) The relative importance of solitary bees and syrphid flies as pollinators of two outcrossing plant species in the New Zealand alpine. Austral Ecol. 38: 169–176. 3. Campbell AJ., Biesmeijer JC., Varma V., Wakers FL. (2012) Realising multiple ecosystem services based on the response of three beneficial insect groups to floral traits and trait diversity. Basic and Appl. Ecol. 13: 363–370. 4. Stokl J.,Brodmann D., Hansson A. (2011) Smells like aphids: orchid flowers mimic aphid alarm pheromones to attract hoverflies for pollination. Proc. R. Soc. B 278: 1216–1222. |
| *Neuroptera larvae*  (lacewings) | Lepidopteran larvae (o, nubialis), aphids.  Spider mite Tetranychus urticae. eggs of Ephestia kuehniella | 1. Romeis J., Dutton A., Bigler F. (2004) Bacillus thuringiensis toxin (Cry1Ab) has no direct effect on larvae of the green lacewing *Chrysoperla carnea* (Stephens) (Neuroptera: Chrysopidae). J Insect Physiol. 50: 175–183. 2. Dutton A., Klein A., Romeis J., Bigler F. (2003) Prey-mediated effects of *Bacillus thuringiensis* spray on the predator Chrysoperla carnea in maize. Biol. Control 26(2):209-215. 3. Meissle M., Zund J., Waldburger M., Romeis J. (2014) Development of *Chrysoperla carnea* (Stephens) (Neuroptera: Chrysopidae) on pollen from Bt-transgenic and conventional maize. Sci. Rep. 4:5900. |
| *Heteroptera*  *Nabis adults and larvae*  (damsel bugs) | Predators of aphids, moth eggs, and small caterpillars, including corn earworm, European corn borer, and some armyworms. Other prey may include small sawfly larvae, mites, tarnished plant bug nymphs, ladybird larvae. | 1. Coll M., Bottrell DG. (1995) Predator-prey association in mono- and dicultures: Effect of maize and bean vegetation. Agric. Ecos. Env. 54(1):115-125. 2. Braman SK. (2000) Damsel bugs (Nabidae). Pp. 639–656. In: Schaefer C. W. & Panizzi A. R. (eds.): Heteroptera of Economic Importance. CRC Press, Boca Raton. 3. Munkvold GP., Hellmich RL. (1999) Genetically modified insect resistant corn: Implications for disease management. APSnet Features. Online. doi: 10.1094/APSnetFeature-1999-1199. 4. Albajes R., Lumbierres B., Pons X. (2011) Two heteropteran predators in relation to weed management in herbicide-tolerant corn. Biol. Control 59: 30-36. 5. Albajes R., Farinós GP., Pérez-Hedo M., Poza M., Lumbierres B., Ortego F., Pons X., Castañera P. (2012) Post-market environmental monitoring of Bt maize in Spain: Non-target effects of varieties derived from the event MON810 on predatory fauna. Spanish J Agric Res. 10(4): 977-985. |
| *Heteroptera*  *Orius adult and larvae*  (minute pirate bug) | Feed mostly on aphids and spider mites, thrips, and their eggs. Also consume lepidopteran eggs. | 1. Michael PS., Lundgren JG. (2010) Oviposition response by *Orius insidiosus* (Hemiptera: Anthocoridae) to plant quality and prey availability. [Biol. Control](http://www.sciencedirect.com/science/journal/10499644) [55(3)](http://www.sciencedirect.com/science/journal/10499644/55/3): 174–177. 2. Hérard F. (1986) Annotated list of the entomophagous complex associated with pear psylla, Psylla pyri (L.) (Hom.: Psyllidae) in France. Agronomie 6: 1–34. 3. Kiman ZB., Yeargan KV. (1985) Development and reproduction of the predator Orius insidiosus (Hemiptera: Anthocoridae) reared on diets of selected plant material and arthropod prey. Annals Entomol. Soc. America 78: 464–467. 4. Justine JL. (1978) Contribution to the study of the influence of alimentation on the development and reproduction of Orius niger Wolff, 1811 (Heteroptera: Anthocoridae). Masters Thesis, University of Montpellier, Montpellier, France, 92 pages. 5. Al Deeb MA., Wilde GE., Higgins RA. (2001) No effect of Bacillus thuringiensis and Bacillus thuringiensis on the predator *Orius insidiosus* (Hemiptera; Acanthocoridae). Environ. Entomol. 30, 625–629. |
| *Araneae*  (spiders) | Prey on aphids, heteropterans and thrips, but also other arthropod groups such as beetles, flies and hymenopterans. | 1. Meissle M., Romeis J. (2009) The web‐building spider *Theridion impressum* (Araneae: Theridiidae) is not adversely affected by Bt maize resistant to corn rootworms. Plant Biotechnology Journal 7(7):645 – 656. 2. Řezac M., Pekar, S., Kocourek F. (2006). Effect of Bt-Maize on Epigeic Spiders (Araneae) and Harvestmen (Opiliones). Plant Protect. Sci. 42: 1–8. 3. Guerrero SP., Gelan-Begna, A., Villa AR., Tamajón R., Vargas-Osuna E. (2014) Lethal and sublethal effects of commercial insecticides on *Philodromus buxi*, a potential predator of defoliating Lepidoptera in woodland in southern Spain, International J Pest Manag. 60:2 121. 4. Romeis J., Meissle M., Álvarez-Alfageme F., Bigler F., Bohan DA., Devos Y., Malone LA., Pons X., Rauschen S. (2014) Potential use of an arthropod database to support the non-target risk assessment and monitoring of transgenic plants, Transgenic Res.23: 6, 995. 5. Schmidt JM., Peterson JA., Lundgren JG., Harwood JD. (2013) Dietary supplementation with pollen enhances survival and Collembola boosts fitness of a web-building spider. Entomol. Exp. Appl. 149, 3. |
| *Aeolothripidae*  (predatorsy thrips) | Other phytophagous thrips and spider mites. | 1. Obrist LB., Klein H., Dutton A., Bigler F. (2005) Effects of Bt maize on *Frankliniella tenuicornis* and exposure of thrips predators to prey-mediated Bt toxin. Entomol. Exp. Appl. 115(3). 409-416. 2. Zwahlen C, Nentwig W, Bigler F, Hilbeck A. (2000) Tri-trophic interactions of transgenic *Bacillus thuringiensis* corn, *Anaphothrips obscurus* (Thysanoptera; Thripidae), and the predator Orius majusculus (Heteroptera; Antho-coridae). Environ. Entomol. 29, 846–850. 3. Obrist LB., Klein H., Dutton A., Bigler F. (2006) Assessing the Effects of Bt Maize on the Predatory Mite *Neoseiulus cucumeris*. Exp. Appl. Acarol. 38(2): 125-139. 4. Jorge B. Torres AE., John R. (2008) Ruberson Interactions of *Bacillus thuringiensis* Cry1Ac toxin in genetically engineered cotton with predatory heteropterans. Transgenic Res. 17:345–354. |

**A. Entry 2**

Coleoptera and Lepidoptera resistant

**S = 32**

**L = 82**


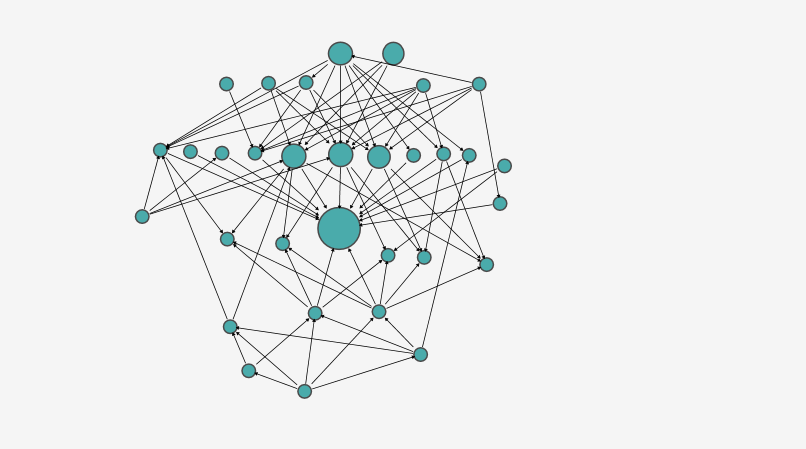


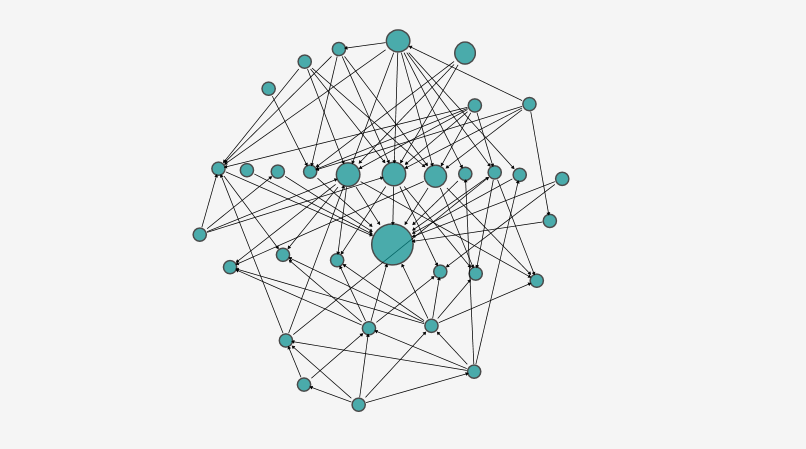


**B. Entry 5**

Lepidoptera resistant and glyphosate tolerant

**S = 33**

**L = 88**


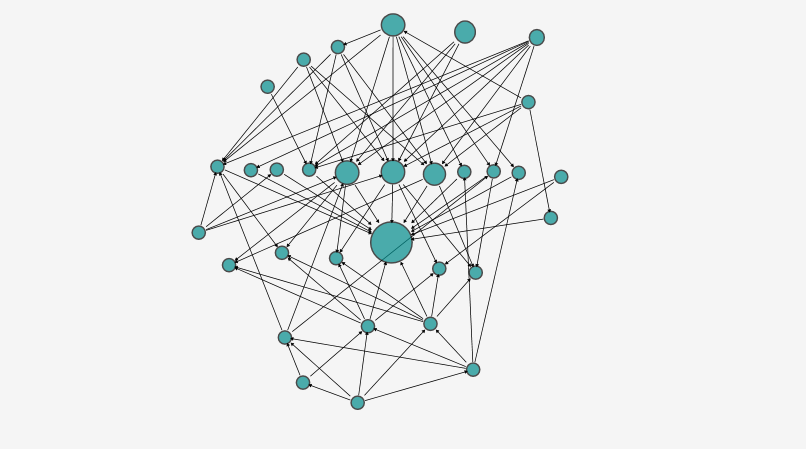


**C. Entry 6**

Lepidoptera resistant and glyphosate tolerant+ glyphosate treatment

**S = 32**

**L = 85**

**D. Entry 7**

Coleoptera and, Lepidoptera resistant and glyphosate tolerant

**S = 32**

**L = 91**


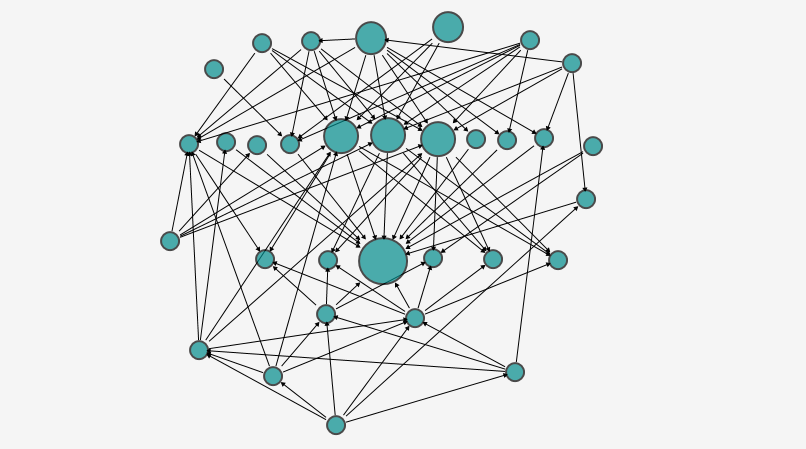


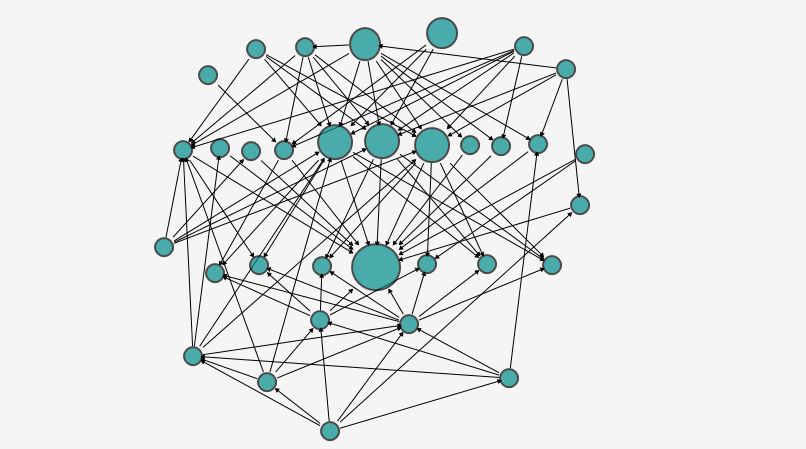


**E. Entry 8**

Coleoptera, Lepidoptera resistant and glyphosate tolerant + glyphosate treatment

**S = 33**

**L = 95**

**F. Entry 901**

Isogenic control

PR-36B08

**S = 30**

**L = 91**


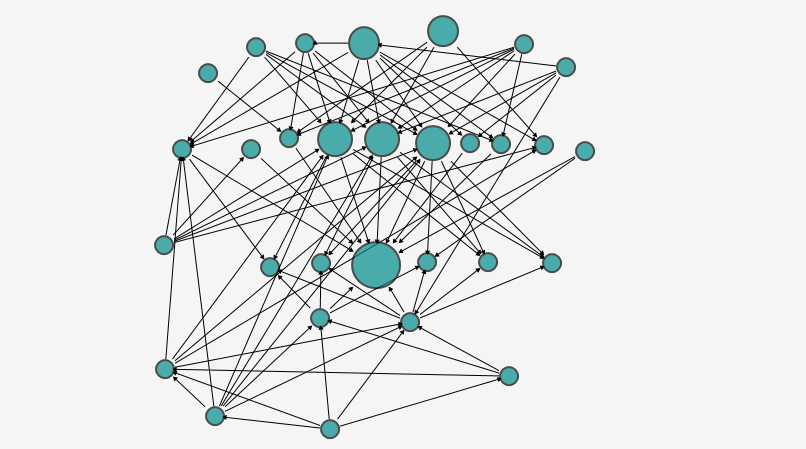


**Figures S2 A-G.** Food-webs on GM and non-GM maize crops. S – number of trophic groups, L – number of links between trophic groups. Food webs were built with CoSBiLab software (Jordán et al. 2012). The position and size of the circles are similar with Entry 1 (Figure 1A, B) and intended to allow an easier way to identify functional groups.
